# Omental infarction in the postpartum period: a case report and a review of the literature

**DOI:** 10.1186/1752-1947-4-368

**Published:** 2010-11-17

**Authors:** Michael Tachezy, Rainer Grotelüschen, Florian Gebauer, Andreas H Marx, Jakob R Izbicki, Jussuf T Kaifi

**Affiliations:** 1 Department of General, Visceral and Thoracic Surgery, University Medical Center Hamburg-Eppendorf, Martinistraße 52, 20246 Hamburg; 2 Institute of Pathology, University Medical Center Hamburg-Eppendorf, Martinistraße 52, 20246 Hamburg, Germany

## Abstract

**Introduction:**

Omental infarction is a rare and often misdiagnosed clinical event with unspecific symptoms. It affects predominantly young and middle aged women.

**Case presentation:**

This is a case report of a 26-year-old Caucasian woman with spontaneous omental infarction two weeks after normal vaginal delivery.

**Conclusion:**

Omental infarction is a differential diagnosis in the postpartum acute abdomen. As some cases of omental infarction, which are caused by torsion, can be adequately diagnosed via computed tomography, a conservative treatment strategy for patients without complications should be considered in order to avoid any unnecessary surgical intervention.

## Introduction

Omental infarction is a rare clinical event that affects predominantly young and middle aged women [1]. It is usually caused by omental torsion, but the reasons for this remains poorly understood. Omental infarction was first reported in 1882 by Oberst [2]. Patients present symptoms of an acute abdomen. The clinical findings are very unspecific and, therefore, in most cases it is surgical exploration that leads to the diagnosis.

This report highlights the case of a spontaneous omental infarction in a young woman in the postpartum period.

## Case presentation

A 26-year-old Caucasian woman presented with a five day history of increasing epigastric pain and nausea two weeks after the vaginal delivery of a healthy child of normal weight and size.

Physical examination revealed a normal peristalsis and supraumbilical tenderness. A small umbilical hernia (< 1 cm diameter), with no signs of incarceration, was described by the initial examining physician. Pulse and blood pressure were normal (85 beats/min, 123/83 mmHg). She was apyrexial but adynamic, with pale and clammy skin. In summary, the general status of the patient was impaired on admission (American Society of Anesthesiologists score 2-3).

Blood tests revealed an elevated white blood cell count (14.7/nL) and serum C-reactive protein (120 mg/dL). A coagulation study (international normalised ratio, partial thromboplastin time, fibrinogen and platelet count) revealed no abnormalities.

Abdominal ultrasound showed no specific pathological findings and, for further clarification, a contrast-enhanced abdominal computed tomography (CT) was performed. The morphologic findings of the CT were interpreted as an incarcerated umbilical hernia by the radiologist. However, due to the clinical presentation of an acute abdomen and the elevated inflammatory blood parameters, the patient was asked to consent to an exploratory laparotomy. A small laparotomy (5 cm long midline incision around the umbilicus) was performed. Contrary to the CT findings, and in accordance to the clinical examination, no umbilical hernia could be detected intraoperatively. Surprisingly, a hemorrhagic greater omentum measuring 11 × 7.5 × 2.5 cm was discovered and resected. A small amount of sanguinous ascites was also found. On further exploration we found no adhesions or other underlying causes for the infarction, such as an external or internal hernia or a vascular pedicle.

**Figure 1:**
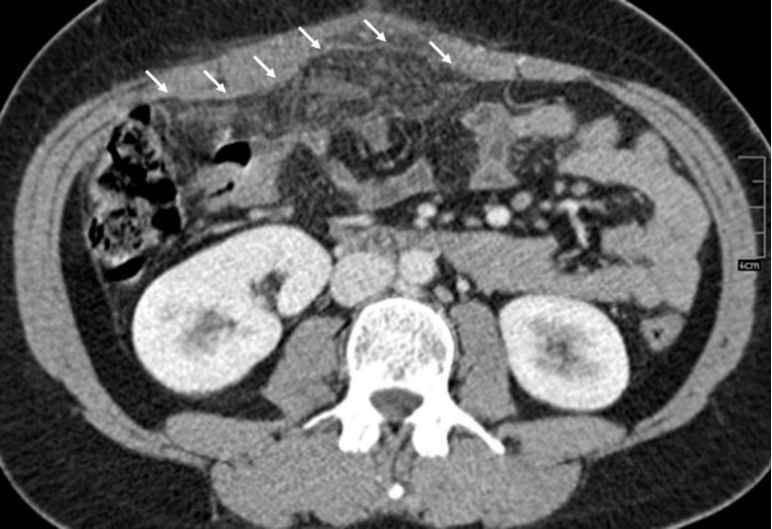
**Computed tomography scan of the abdomen showing a hypoperfused mass in the anterior portion of the median epigastrium with fatty density (→) and a thin layer of free fluid surrounding the liver**.

In a retrospective repeat analysis of the CT scan, a hypoperfused mass of fatty appearance in the anterior portion of the midabdomen and small amounts of free fluid surrounding the liver were observed (Figure 1).

**Figure 2:**
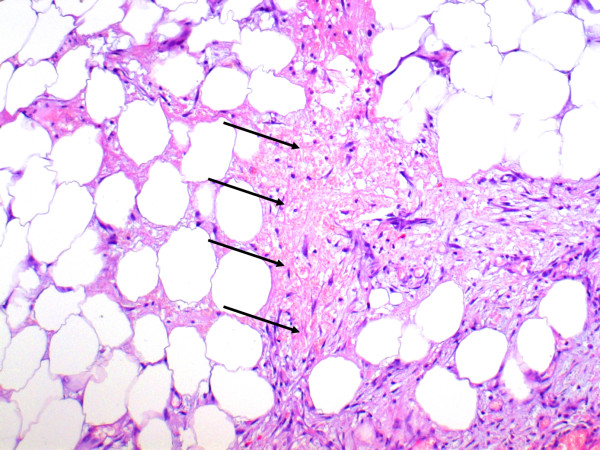
**Histological findings of omentum majus show fresh hemorrhagic circulation disorders (arrows), partial necrosis of fatty tissue with acute inflammatory cell infiltrate (hematoxylin staining, original magnification × 100)**.

Histopathological findings of the resected omental specimen confirmed fresh hemorrhagic infiltrations of the tissue, partial thrombosis of the small vessels and, in some parts, necrotic fatty tissues with an acute inflammatory cellular infiltrate (Figure 2). Further laboratory testing excluded potentially underlying coagulopathy or rheumatic disease.

The patient was discharged after an uneventful recovery three days after surgery.

## Discussion

Omental infarction was first described in the late 19th century and, since then, only a few hundred cases have been published in the English literature [3]. This is one of the first cases showing spontaneous omental infarction in the puerperium after a vaginal birth. Two previously published cases describe omental infarction in the postpartum period - one after caesarean section and another after vaginal delivery [4,5]. Torsion of the omentum is the main reason for infarction and two different forms have been described: primary torsions (without other pathologic intraabdominal findings) and secondary torsions (tumors, cysts, inflammatory changes, adhesions, hernias). Predisposing factors for torsion are anomalies of the omentum, such as a small root, irregular vascular anatomy, abdominal trauma, cough and physical strain [2].

The etiology of omental infarction without torsion remains uncertain but several mechanisms have been proposed, such as an anomaly of venous vessels [6]. Other possible causes for primary infarctions could be disorders of hemostasis or vascular diseases. It is known that hematologic changes occur during pregnancy and the puerperium and that hypercoagulability leads to an increased risk of thromboembolic events [7]. The exact mechanism leading to infarction in this case remains unclear. Possible changes during the return of the mother's body to the pre-pregnancy physiological condition may have provoked the infarction. Usually the clinical symptoms of an infarction of the omentum are localized peritoneal irritation on the right side of the abdomen, sometimes associated with low-grade fever. As in the present case, the C-reactive protein and white blood count may be elevated. The clinical picture often misleads physicians to assume an incorrect preoperative diagnosis such as acute cholecystitis, appendicitis, diverticulitis, appendicitis epiploica or umbilical hernia [3,8,9].

As most patients show symptoms of an acute abdomen, CT of the abdomen and pelvis should be the diagnostic imaging of choice [10]. If omental infarction is caused by torsion, characteristic CT-findings might be detectable. The torsion leads to the presence of concentric linear strands in the fatty mass, a so-called 'fat spiral pattern' [11]. In our case no omental torsion was present and, consequently, the radiologist was unable to identify this diagnostic radiologic sign. Therefore, differentiating the omental infarction from other abdominal or omental diseases was challenging and the radiological findings were misinterpreted as a small incarcerated umbilical hernia.

Diagnosis of an omental infarction has traditionally been made intraoperatively during an exploratory laparotomy or laparoscopy and the treatment has been partial or total omentectomy. Recent reports highlight cases of patients with CT diagnosed omental torsions who have been successfully treated conservatively without any other complications (such as bacterial superinfections) [12-15]. Whenever conservative treatment fails, or the clinical status of the patient worsens, a surgical intervention should be quickly implemented.

## Conclusion

Omental infarctions are often not initially considered in the differential diagnosis of a post partum acute abdomen. When omental infarction is caused by torsion, a correct preoperative diagnosis by contrast-enhanced CT scanning can avoid surgery. Recently published case series have reported successful conservative management.

## Competing interests

The authors declare that they have no competing interests.

## Authors' contributions

MT, RG and JTK managed the patient and reviewed the literature. MT and RG were the main authors of the manuscript. AHM analyzed the histopathological specimen. FG, JTK and JRI made modifications to the manuscript. All authors read and approved the final manuscript.

## Consent

Written informed consent was obtained from the patient for publication of this case report and any accompanying images. A copy of the written consent is available for review by the Editor-in-Chief of this journal.
